# Feasibility of ultrasound measurement in a human model of acute compartment syndrome

**DOI:** 10.1186/s13017-019-0222-9

**Published:** 2019-02-04

**Authors:** Jakob Mühlbacher, Reinhard Pauzenberger, Ulrika Asenbaum, Tobias Gauster, Stephan Kapral, Harald Herkner, Andreas Duma

**Affiliations:** 1 Department of Surgery, Division of General Surgery, Medical University of Vienna, Waehringer Guertel 18-20, A-1090 Vienna, Austria; 2 Department of Surgery, Division of Plastic and Reconstructive Surgery, Medical University of Vienna, Vienna, Austria; 3 Department of Biomedical Imaging and Image-guided Therapy, Medical University of Vienna, Vienna, Austria; 4 Institute of Applied Statistics and Computing, University of Natural Resources and Life Sciences, Vienna, Austria; 5 Department of Anaesthesiology and Intensive Care, AUVA, Linz, Austria; 6 Department of Emergency Medicine, Medical University of Vienna, Vienna, Austria; 7 Department of Anaesthesiology and General Intensive Care, Medical University of Vienna, Vienna, Austria

**Keywords:** Acute compartment syndrome, Ultrasound, Lower extremity, Human cadaver

## Abstract

**Background:**

Early diagnosis of acute compartment syndrome (ACS) of the leg is essential to improve the outcome. Direct invasive measurement is currently recommended to measure intracompartmental pressure. A non-invasive and reproducible means of making the diagnosis would be a step forward. The purpose of this exploratory study was to investigate the feasibility of non-invasive ultrasound-guided angle measurement as a surrogate of increased pressure in a model of ACS.

**Methods:**

A model of ACS was generated by infusion of saline into the anterior compartment of the leg of human cadavers to incrementally increase the intracompartmental pressure from 10 to 100 mmHg. In 40 legs (20 cadavers), the angle (TFA, tibia-fascia angle) between the anterolateral cortex of the tibia and the fascia of the anterior compartment was measured at each 10 mmHg pressure increase using ultrasound in a standardized transversal plane. A multilevel linear regression model was used to estimate intracompartmental pressure from delta TFA (ΔTFA).

**Results:**

TFA (mean [± SD]) increased from 61.0° (± 12.0°) at 10 mmHg up to 81.1° (± 11.1°) at 100 mmHg compartment pressure. Each increase ΔTFA by one degree was associated with an increase in pressure by 3.9 mmHg (95% CI, 3.8–4.0, *p* < 0.001).

**Conclusions:**

We found that intracompartmental pressure of the anterior compartment of the calf can be well estimated by ultrasound-based ΔTFA in this post mortem experiment. Our findings indicate that non-invasive TFA measurement is feasible and it is reasonable that this will hold true in real life, but the findings are too preliminary to be used in clinical practice now.

## Introduction

Acute compartment syndrome (ACS) of the lower extremity is a condition of rapidly increasing pressure leading to reduced perfusion below a vital level for muscles and nerves within limited anatomic space [1–5]. Most frequently, ACS results after severe traumatic leg injuries [6, 7]. However, other causes such as arterial injury, vascular occlusion, crush injury or contusion while in an anticoagulation state have been described [8]. Without immediate surgical decompression, nerve lesions, muscle contractures, amputation or even sepsis may occur [9, 10]. Therefore, early diagnosis is essential to avoid irreversible damage [4, 11–15]. Currently diagnosis is based on physical examination [11–14]. Invasive pressure measurement is recommended as an adjunct to clinical examination [13, 14]. Non-invasive alternatives such as near-field spectroscopy, microvascular blood flow, muscle oxygenation and pH, laser Doppler flowmetry, quantitative hardness measurements or compression sonography have been examined but were not feasible for routine diagnosis of ACS [15–21]. Ultrasound examination is non-invasive, easy to perform, painless and could be used in addition to invasive pressure measurement [22–25].

We hypothesized that increasing pressure following tissue expansion will shift the fascia away from the plane anterolateral cortex of the tibia in the anterior compartment. So far, the compartment width was evaluated using ultrasound to detect changes in healthy volunteers following exercise [22, 23]. Shifting of the angle between the tibia and the muscle fascia may be visible and quantifiable with 2-D ultrasound. In this exploratory human cadaver study, ultrasound was used to estimate pressure in the anterior compartment of the lower extremity based on changes of TFA (tibia-fascia angle).

## Materials and methods

### Study design and objectives

After local institutional review board approval, an exploratory study in human cadavers was performed. Unmodified adult cadavers, within 24 h after death, were included. Based on physical examination and medical records, specimen with compartment syndrome, signs of previous surgery, trophic disorders, injuries at lower extremities or known gait abnormality were excluded. Experiments were performed in 40 legs (20 right and 20 left) of 20 human cadavers to measure TFA in the anterior compartment of the lower extremity over several pressure levels.

### Experiments and measurements

**Fig. 1:**
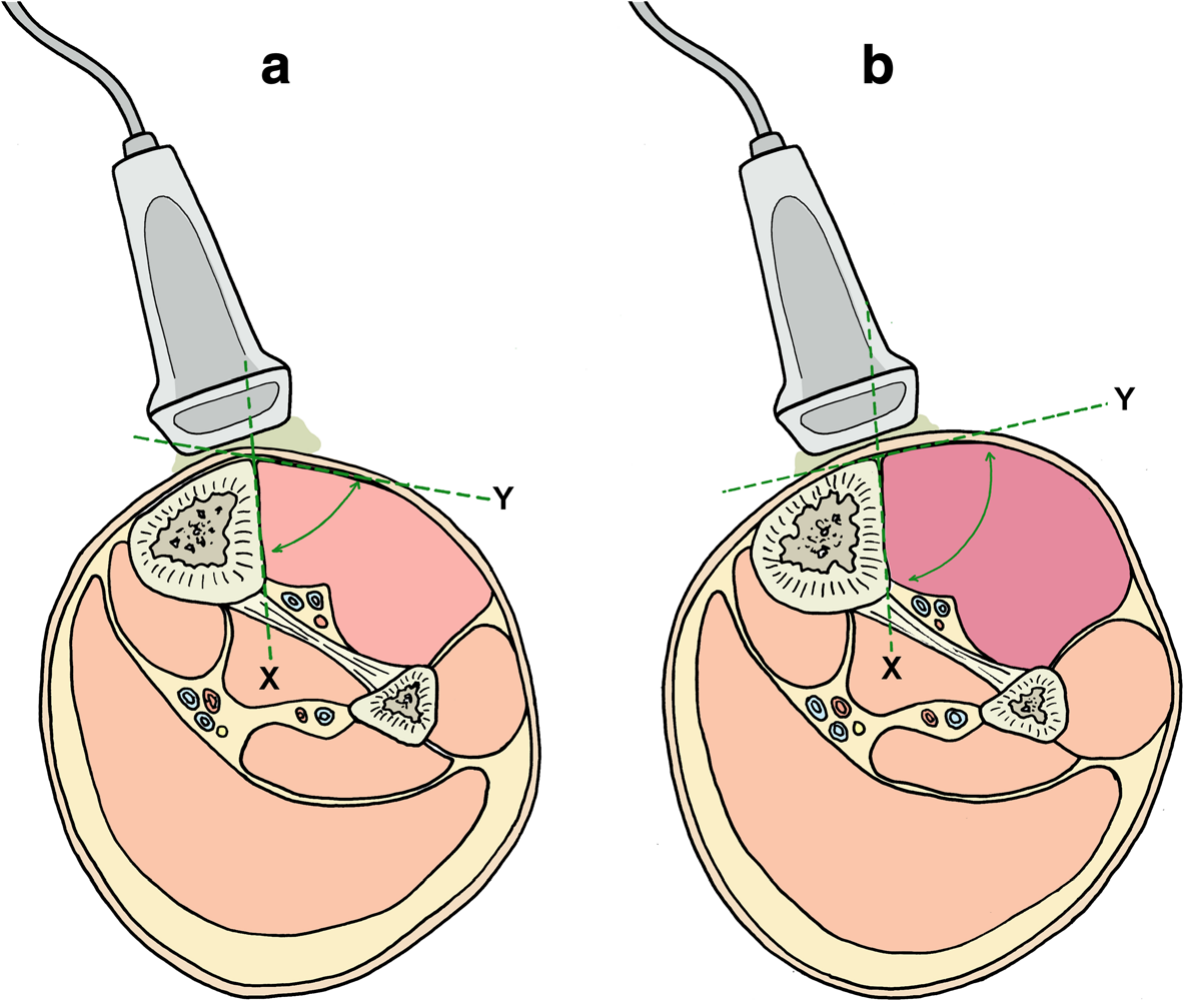
**a**, **b** Schematic illustration of increasing compartment pressure and angle measurement. Schematic illustration of sonographic TFA (tibia-fascia angle) measurement. One line (*X*) was set on the anterolateral cortex of the tibia. The other line (*Y*) was set as a tangent to the curving anterior compartment fascia with its origin at the tibial attachment. TFA was the angle measured between line *X* and *Y*. Measurement under normal conditions (**a**), enlarged angle in the anterior compartment (**b**)

**Fig. 2:**
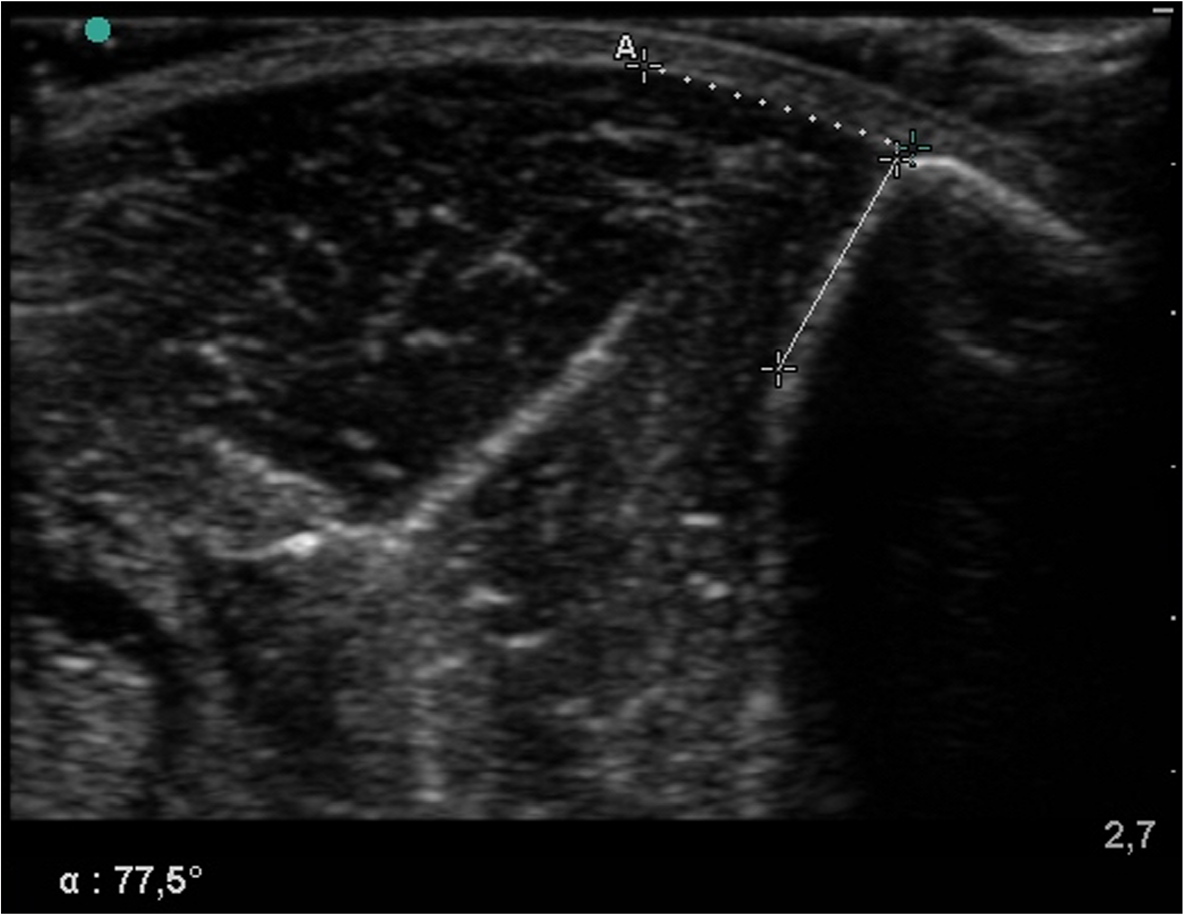
Ultrasound image of one specimen with measurement of TFA (tibia-fascia angle). Ultrasound image of TFA measurement (*male cadaver, right leg*) at a generated pressure level of 40 mmHg. For TFA measurement one line (solid line) was set on the anterolateral cortex of the tibia and the other line (dotted line) was set as a tangent to the curving anterior compartment fascia with its origin at the tibial attachment. TFA was the angle between the two lines (in this case 77.5°)

After collecting base line characteristics, each leg was marked at predefined points for standardized and repeatable measurement of TFA using ultrasound. An ultrasound transducer (Linear Transducer, Typ HFL38/13-6 MHz, MicroMaxx, Fujifilm SonoSite Inc. US, Bothell, Washington, USA) was placed in a 90° angle to the ventral edge of the tibia (Fig. 1 a, b) at the end of the proximal third of the distance from the tibial tuberosity to the intermalleolar line. Five centimeters above and two centimeters lateral to TFA measurement, a 14-gauge cannula was placed into the anterior compartment. This cannula was connected to a P-50 pressure Statham transducer (Gould-Statham Instruments Inc., Bayamon, PR) for continuous compartment pressure monitoring. Signals of the P-50 transducer were amplified using a validated custom instrumentation amplifier (linearity was within ± 3%, zero stability showed ± 1 cmH2O, 12 h observation period). Another 14-gauge needle was inserted five centimeters below and two centimeters lateral to TFA measurement for infusion of 0.9% saline into the anterior compartment to achieve reproducible pressure levels in the area of sonographic evaluation [26]. Lifting a fluid column elevated the hydrostatic pressure and increased the intracompartmental pressure to desired levels [27, 28]. In order to compensate post mortem dehydration and to verify functionality, each leg was initially rehydrated by injecting fluid to raise pressure to 100 mmHg, which was followed by a passive pressure decrease to 10 mmHg [24]. Afterwards, compartment pressure was again increased in increments of 10 mmHg up to 100 mmHg [27, 28]. At each pressure level, the tibia-fascia angle (TFA) was measured by means of real-time ultrasound with constant position of the ultrasonic transducer in transversal plain, respectively. Unmodified ultrasound images were stored for subsequent analysis. For TFA measurement, one line was set on the plane anterolateral cortex of the tibia, the other line as a tangent to the curving anterior compartment fascia with its origin at the tibial attachment of the fascia. TFA was the angle between line *X* and *Y* (Fig. 1 a, b, Fig. 2).

### Statistical methods

Data are presented as mean ± standard deviation (SD) or median and 25–75% IQR for continuous variables and absolute count with a relative frequency for categorical variables. A theoretical cumulative distribution function was fitted to the absolute value of the TFA difference to estimate exceedance probabilities for given quantiles. To access inter-observer variability, 40 (10%) randomly selected (stratified by sex, side, and pressure range) unmodified ultrasound images were analyzed by two independent observers, both experienced in the application of ultrasound; intraclass correlation coefficient (ICC) was calculated. We calculated delta TFA (ΔTFA) as the difference of pressure-specific TFA and TFA to the contralateral leg baseline TFA at 10 mmHg. By study design, we assumed data to be correlated within individuals and within legs resulting in a multilevel structure. To allow for this, we used a multilevel linear regression model to estimate intracompartmental pressure by ΔTFA. The model included subject level (*n* = 20), and leg level (right/left) of each 10 measurements at different pressure levels. We performed sensitivity analysis to assess the robustness of our modeling procedure by using multivariable multilevel linear regression models with potentially confounding covariables, polinomial terms, and multilevel ordinal logistic regression. Statistical analysis and figures were done using R (Version 3.3, https://www.r-project.org, Vienna, Austria) and Stata 14 for Mac (Stata Corp., College Station, TX). Generally, a two-sided *p* value less than 0.05 was considered statistically significant.

### Source and funding

There was no external source of funding for this study.

## Results

**Fig. 3:**
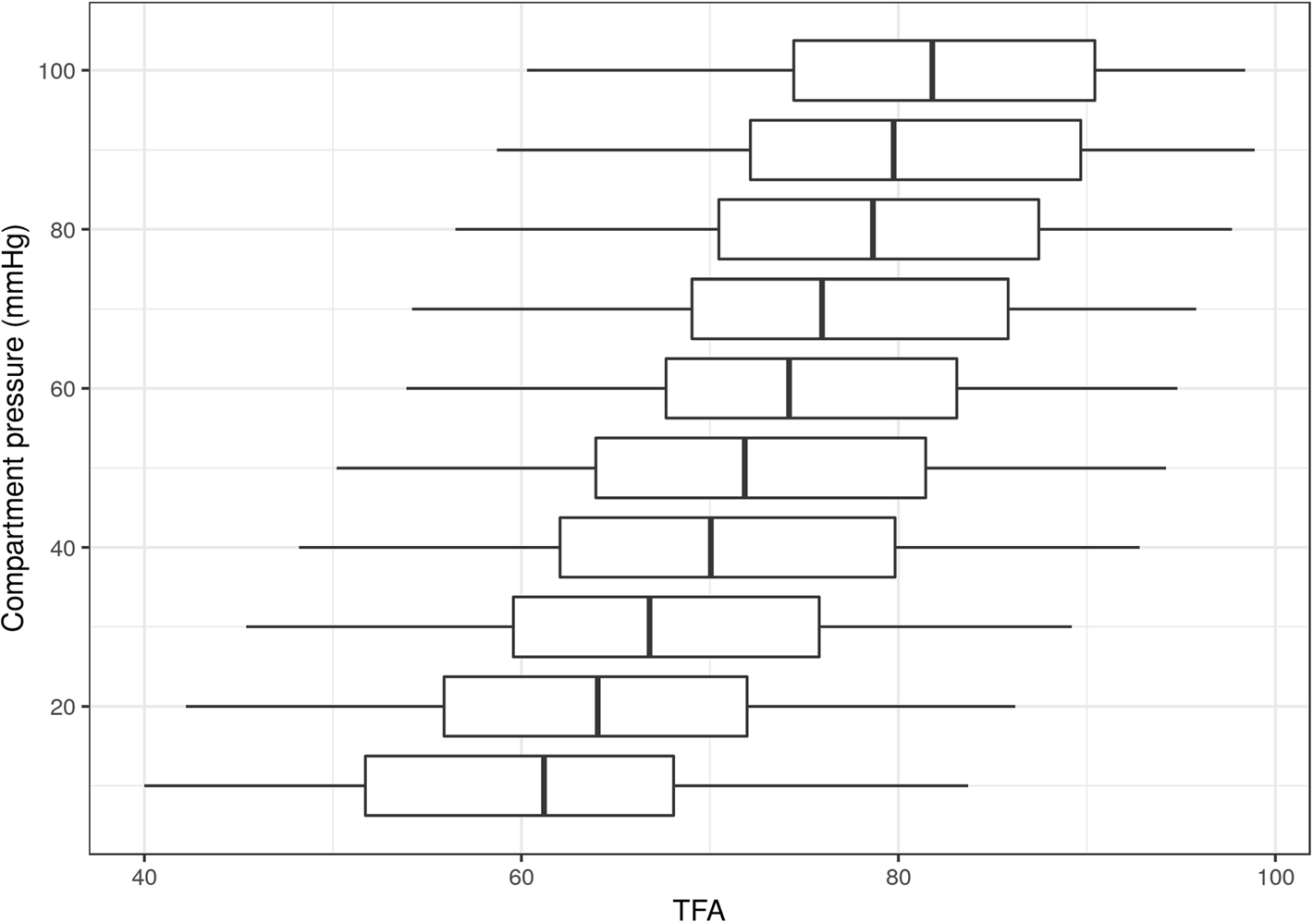
Relation between tibia-fascia angle (TFA) measurements and pressure. Boxplots of TFA measurements at different pressure levels

**Fig. 4:**
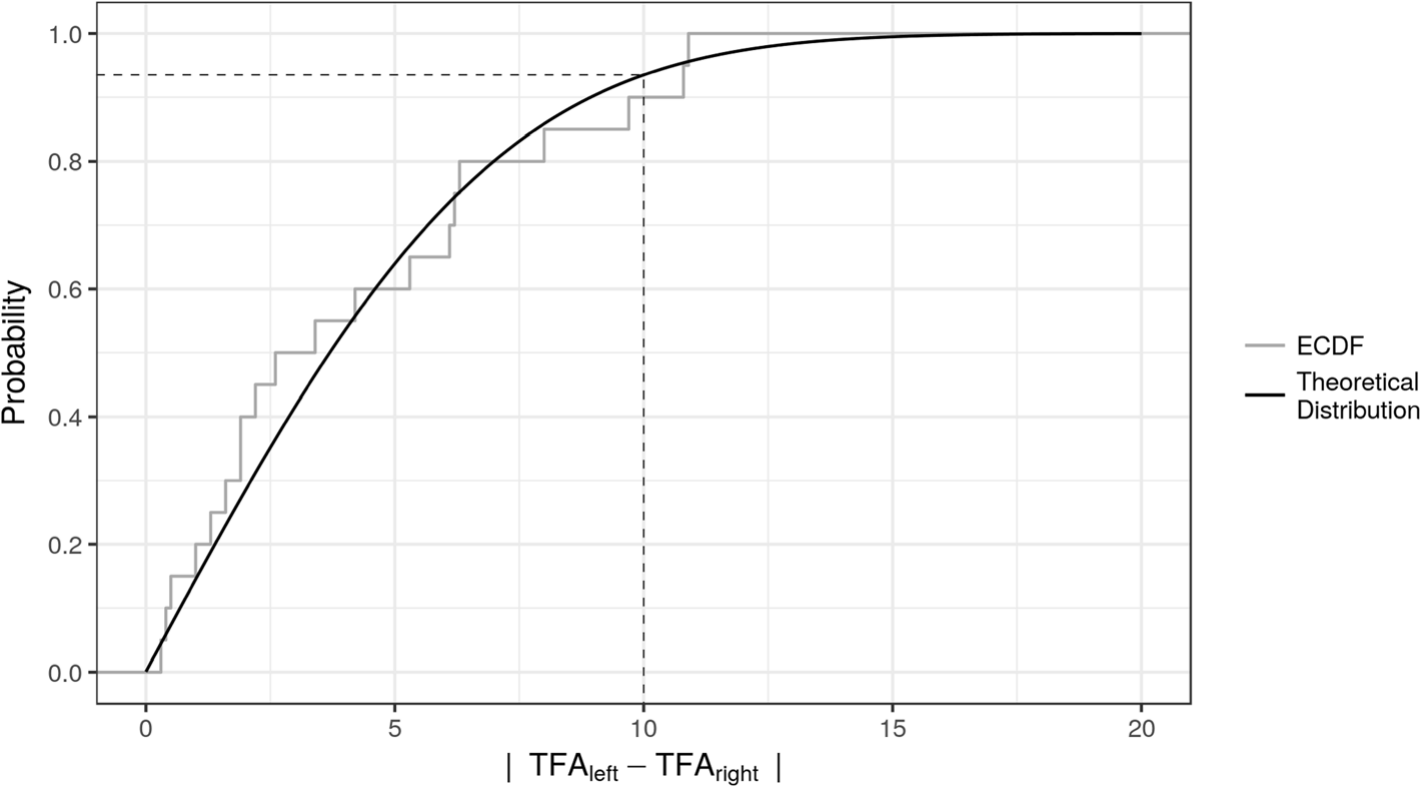
Empirical cumulative distribution function (ECDF) and fitted theoretical distribution of absolute TFA (tibia-fascia angle) differences. ECDF (gray line) for absolute differences of the TFA between legs in each subject at a given 10 mmHg pressure level, which we considered as relevant, and the fitted theoretical distribution function (black line). The dotted line shows given quantiles at an absolute TFA difference of 10° based on the fitted theoretical distribution function

**Fig. 5:**
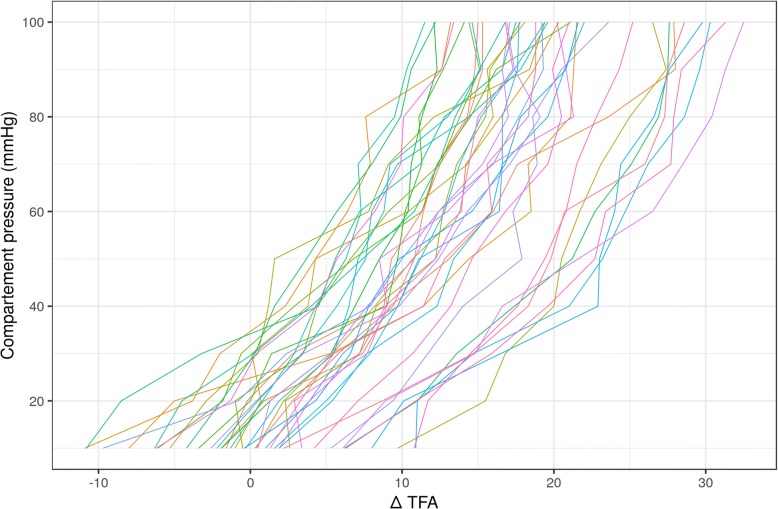
Relation between ΔTFA and pressure for each individual leg (connected line plot)

Baseline characteristics of human bodies included in the exploratory study are detailed in Table 1. Within each observed lower leg compartment (*n* = 40), the measured angle increased with rising pressure. Mean (± SD) TFA rose from 61.0° (± 12.0°) at 10 mmHg to 81.1° (± 11.1°) at 100 mmHg (Fig. 3). The relation between ΔTFA and pressure for each individual leg is presented in Fig. 5 in the “Appendix”. Comparing legs in each individual at a pressure level of 10 mmHg revealed a low probability of 7% for an absolute TFA difference of more than 10° following the theoretical distribution function, whereas it was less than 10° in 93% (Fig. 4). TFA measurement had good inter-observer reliability (ICC 0.77, 95% CI 0.65 to 0.86). Using the multilevel linear model, we found that between 10 and 100 mmHg, a change of one degree in ΔTFA was associated with a pressure increase of 3.9 mmHg (95% CI, 3.8–4.0, *p* < 0.001). Adding age, gender, weight, height, tuberosity-intermalleolar distance and lower leg circumference as covariables to the model left the estimate virtually unchanged. Likewise sensitivity analyses using other modeling strategies indicated robust estimates.

**Table 1:** Demographical data of study subjects

Parameters	All study subjects (*n* = 20)
Baseline data	
Age, years, median (IQR)	81 (71–86.3)
Female sex, *n* (%)	10 (50)
Body mass index, median (IQR)	24.3 (22.4–25.7)
Lower leg circumference^a^, median (IQR)	29 (27–31.5)
Tuberosity—intermalleolar distance^b^, median (IQR)	31 (30–33)

*IQR* interquartile range

^a^Circumference measured on standardized points (cm)

^b^Distance between tuberosity of the tibia and the intermalleolar distance (cm)

## Discussion

In this exploratory study, we investigated the feasibility of ultrasound-guided measurement of TFA, the angle between the rigid anterolateral cortex of the tibia and the fascia, as a surrogate parameter for increased pressure in the anterior compartment of the lower extremity in a human cadaveric compartment syndrome model. Reproducible pressure levels could be generated in the anterior compartments, and sonographic measurement of the TFA was feasible and possible in all of the cadavers despite postmortem alterations.

Our study has several novel findings. We found a robust linear relation between ΔTFA and intracompartmental pressure. If only one leg is affected, this relation can be used to estimate intracompartmental pressure based on ΔTFA. Each degree difference in TFA between the affected leg and the healthy leg is associated with an increase in intracompartmental pressure by approximately 4 mmHg. Second, side-to-side variability in TFA was small, indicating that measuring a difference from the corresponding spot on the other side (if available) may be of value. An absolute difference of > 10° was observed with a low probability of 7% at a pressure of 10 mmHg. Third, interindividual variability in TFA was high, at low as well as elevated pressure levels. For example, at 30 mmHg compartment pressure, TFA varied from 45° in one cadaver to up to 89° in another, at 50 mmHg from 50° to 94° respectively. This variation might be probably due to different body constitution and muscle mass among the small cohort of investigated cadavers. This issue can be easily overcome when the contralateral leg can be used as a reference, as we did in our study.

The anterior leg compartment pressure has important clinical relevance in ACS on its own [10, 26, 29, 30]. So far, ultrasound-guided evaluation of the anterior compartment has only been made in a physiological low-pressure range in healthy volunteers [22, 23]. In this study, we defined only a single position of TFA measurement between the middle and the proximal third of the distance from the tibial tuberosity to the intermalleolar line due to easy accessibility in view of possible further studies, for easy reproducibility, for standardized measurement and in expectation of maximal enlargement of the compartment at this level [22, 23]. Compared to previous experiments, we punctured the compartment 5 cm below and 5 cm above the side of ultrasound-guided measurement for infusion and continuous pressure monitoring to ensure desired intracompartmental pressure levels at the side of TFA assessment [26, 31–33].

This exploratory study has several limitations. Our inclusion was limited to old cadavers (median age of 81 years). They probably had reduced muscle mass, which may have led to systematically smaller TFA and/or different pressure values compared to a cohort of younger age or higher body mass index. Also, generalizability may be limited because this age group rarely suffers traumatic compartment syndrome. However, other causes of ACS at this age have been described [8]. The influence of increased pressure following rehydration of cadavers as well as post mortem alterations on TFA is unknown and may have biased the accuracy of our results [24]. Another limitation is that our investigation is limited to the anterior compartment on one single spot. The plausible relation of TFA of the anterior compartment with the deep posterior compartment pressure has not been tested in this study. Therefore, TFA cannot be used as a surrogate for the pressure in other compartments at this point. Another limitation is that our cadavers had no blood pressure. Therefore, we were not able to determine the confounding effect of blood pressure on TFA. This would be important since diastolic blood pressure may be a variable of interest in the decision-making process for surgical intervention. Furthermore, infusion of saline was used resulting in increased extracellular volume and subsequent pressure increase, whereas clinically compartment syndrome usually occurs due to cellular edema. The contact pressure of the ultrasound transducer on the compartment during measurement may have resulted in underestimation of TFA. Assessment of TFA was done on one standardized longitudinal level only. However, TFA may vary across different levels of the tibia. Pressure patterns along the compartment and in cases of fractured tibia were not investigated in this experimental study and may vary. Despite remarkable advantages, ultrasound is not always available and sonographic assessment is known to be skill and operator dependent. Even though, analysis of inter-observer variability revealed a substantial reliability (ICC 0.77) of this technique, intra-observer variability was not determined in this study. The findings of this exploratory study should not be used to change patient care at this stage; the method is unlikely to be used in clinical practice from now. However, this study should inform future studies in living patients with varying values of intracompartmental pressure.

## Conclusions

We found that intracompartmental pressure of the anterior compartment of the calf can be well estimated by ultrasound-based ΔTFA in this post mortem experiment. Our findings indicate that non-invasive TFA measurement is feasible and it is reasonable that this will hold true in real life, but the findings are too preliminary to be used in clinical practice now.

## Abbreviations

ACS: Acute compartment syndrome
CI: Confidence interval
ECDF: Empirical cumulative distribution function
ICC: Intraclass correlation coefficient
IQR: Interquartile range
SD: Standard deviation
TFA: Tibia-fascia angle
ΔTFA: Delta tibia-fascia angle
